# Shift Work and Dry Eye Disease in the Korean Working Population: A Population-Based Cross-Sectional Study

**DOI:** 10.3390/ijerph18105492

**Published:** 2021-05-20

**Authors:** Joonho Ahn, So-Jung Ryu, Jihun Song, Hyoung-Ryoul Kim

**Affiliations:** 1 Department of Occupational and Environmental Medicine, Seoul St. Mary’s Hospital, College of Medicine, The Catholic University of Korea, Seoul 06591, Korea; drcox@naver.com (J.A.); ifyshine@gmail.com (J.S.); 2 Department of Ophthalmology, Taereung Bright Eye Clinic, Seoul 01858, Korea; skysojung@nate.com

**Keywords:** shift work schedule, ophthalmology, dry eye syndromes, age groups

## Abstract

This study aimed to evaluate the association between shift work and dry eye disease (DED) in the general population. The 2011 Korea Health Panel (KHP) was used. Chi-square test and multivariate logistic regression were used to assess the relationship between shift work and DED. Stratification analysis was conducted by sex and age. Overall, the odds ratio (OR) of DED according to shift work did not showed significant results (adjusted OR = 1.230, 95% CI 0.758–1.901). When findings were stratified based on age older or younger than 40 years, the OR of DED increased to 2.85 (95% CI: 1.25–5.90) in shift workers under 40 years of age. Our results show an association between shift work and DED in a group of younger subjects.

## 1. Introduction

Dry eye disease (DED) has a high prevalence among chronic eye diseases, although differences are reported in existing research [1,2]. As defined at the Tear Film & Ocular Surface Society Dry Eye Workshop II (TFOS DEWS II) in March 2015, DED is a multifactorial disease of the ocular surface characterized by a loss of homeostasis of the tear film, and accompanied by ocular symptoms, in which tear film instability and hyperosmolarity, ocular surface inflammation and damage, and neurosensory abnormalities play etiological roles [3]. Dry eye disease (DED) is a significant chronic disease in terms of quality of life because it can cause foreign body sensation, pain, sleep disorders, and mental disorders, together with potentially affecting work productivity [4,5].

According to previous studies, major risk factors for DED include older age, female, postmenopausal estrogen therapy, a history of ocular surface surgery, and antihistamine medications [6]. Other studies have reported occupational risk factors. Visual display terminal syndrome, which is caused by looking at a computer monitor for a long time, is known to be related to dry eye [7]. In survey-based research on DED in tropical countries, outdoor environments, sunlight, and air pollution have been reported to be occupational hazards [8].

Recently, an association between night shift work and dry eye syndrome has emerged in research [9]. Although reports differ, about 20% of workers worldwide perform shift work [10,11]. Shift work affects circadian rhythm and certain lifestyle aspects, such as exercise and eating habits, and it can cause various diseases [12]. Shift work is associated with metabolic risk factors such as hypertension, diabetes, and obesity, as well as risk of sleep disturbance and cardiovascular disease [13,14,15]. There is only one study on the risk of DED in shift work, however, and that study included a small sample of only 50 subjects [9].

The present study aims to identify the relationship between shift work and dry eye syndrome, proposing a population-based study to overcome the limitations of previous research. Additionally, sex and age are known major risk factors for DED [16,17]. Because these variables are likely as effect modifiers, we explored further stratification analysis.

## 2. Materials and Methods

This study is compliant with methods detailed in the statement on Strengthening the Reporting of Observational Studies in Epidemiology (STROBE) [18].

### 2.1. Study Participants

Our study is based on data obtained from the Korea Health Panel (KHP), which comprises nationally representative datasets of the Korean general population, and all of the study designs are stratified, multi-stage cluster samples conducted by Korea Institute for Health and Social Affairs. Content is derived from nationwide cluster sampling based on the 2005 Population and Housing Census. Some survey contents vary by year, and the only recent year that included codes for both shift work and DED was 2011. Thus, we used data from 2011.

**Figure 1:**
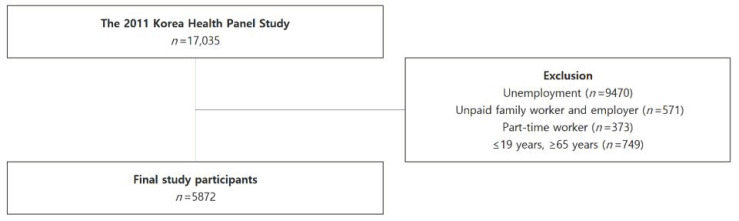
Schematic diagram of study participants.

A total of 17,035 people was originally surveyed in the 2011 KHP data (Figure 1). Because this study focuses on full-time adult paid workers, we excluded subjects with an unemployment status (*n* = 9470), unpaid family workers and employers (*n* = 571), part-time workers (*n* = 373), and workers aged ≤19 or ≥65 years (*n* = 749). The final number of study participants was 5872.

### 2.2. Main Exposures and Outcomes

For the purpose of analysis in this study, shift work was an independent variable and dry eye disease was a dependent variable. Shift work was identified among subjects based on the answer to the question, “As of 31 December 2010, did you mainly work during the day (between 6 a.m. and 6 p.m.)? Or did you work during a different time?” If a participant answered, “I worked mainly during the day”, then the participant was defined as a non-shift worker; all other subjects were defined as shift workers. The surveyor investigated the use of outpatient services through hospital receipts and National Health Insurance Service (NHIS) data to determine diagnosis names and diagnosis codes. Accordingly, DED was defined as any case wherein the diagnosis name of dry eye disease was identified.

### 2.3. Covariate

Referring to previous research in which DED is shown to have increased in subjects aged 40 years or older, findings were stratified using the age of 40 years [2]. We identified subjects’ disease history of diabetes mellitus, connective tissue disease, acne, and gout by investigating these specific diagnosis names and diagnosis codes. In response to the question about smoking history, if participants answered that they “currently smoke every day” or smoke “sometimes”, they were defined as current smokers. If participants answered that they had “smoked in the past but do not currently smoke”, they were defined as ex-smokers. Patients reporting a smoking history of “None” were defined as non-smokers. In response to the question about the use of alcohol, participants who answered that they had a history of “not drinking for life” or “not drinking for the last year” were classified as “no” for alcohol drinking. Other answers classified participants as “yes” for alcohol drinking.

### 2.4. Statistical Analysis

According to DED, the characteristics of the study population were analyzed using the χ2 test. Moreover, multivariate logistic regression was implemented to analyze the relationship between shift workers and DED by calculating odds ratio (OR) and 95% confidential interval (CI) and adjusting for risk factors such as connective tissue disease, smoking, alcohol use, acne, and gout. Age group and sex were used as stratification variables. Though there are many known risk factors for dry eye syndrome, the major risk factors are age and sex [16,17]. These two confounders were not simply adjusted, but stratified, which showed differences in effect sizes and interactions among subgroups. Since age may have an effect on DED within each age group even after stratification, adjustment for age was also performed in each age group. In most societies, the normal retirement age is 65 years [19]. Therefore, we surveyed populations excluding those 65 or over, like other studies in workers [20]. For sensitivity analysis, we conducted the same analysis in a study population including participants 65 years of age or older. Data were analyzed using SAS 9.4 software (SAS Institute Inc., Cary, NC, USA).

## 3. Results

### 3.1. Characteristics of Participants

Table 1 shows the characteristics of participants according to DED. Of the total participants, the percentage of those diagnosed with DED was 3.08%. In comparison to the non-DED group, the DED group had a significantly larger proportions of older people (aged 40 years or over), women, smokers, and people with diabetes mellitus. However, there was no significant difference between the two groups according to work schedule, occupational classification, alcohol use, connective tissue disease, acne, or gout.

### 3.2. Association between Shift Work and DED

Table 2 reports the results of the univariate and multivariate analyses in detail with odds ratio and 95% CI. In the overall population, no significant differences were found between shift workers and DED (adjusted OR = 1.202, 95% CI 0.741–1.860). In stratification based on age of 40 years, shift workers younger than 40 (adjusted OR = 3.061, 95% CI 1.336–6.395) showed a statistically significantly increased risk of DED, unlike in the 40 years and older group (adjusted OR = 0.923, 95% CI 0.490–1.598). The *p*-value for the effects of interaction between age group and shift work on DED was 0.037. In contrast, there was no significant difference in the analysis stratified by sex.

Additionally, sex-/age-group-stratified analysis was performed (Table A1). Both the female group and the male group showed differences in OR values according to age groups, but the *p*-value for the interaction test was only statistically significant in the female group. As a sensitivity analysis, an analysis study population including those aged 65 years or older was used to calculate the OR (Table A2). The sensitivity analysis results were similar to those in the analysis of the study population that excluded people aged 65 years or older due to retirement from the workforce.

## 4. Discussion

In summary, unlike in the overall population, shift workers under 40 years of age showed significantly higher risk of DED. The work of Ali Makateb et al. showed that shift work can cause tear film instability and exacerbation of dry eye symptoms [9]. That research shows significant results in the overall population. Unlike our study, however, that research does not show results of stratification analysis according to age. The difference of these results is presumed to be due to the fact that their overall population itself in the work of Ali Macateb et al. is a young group. The population in the work of Ali Macateb et al. was 24–50 years old, with a mean age of 33.34 ± 6.5 years, which is similar to that of the younger subgroup in the analysis herein. Regarding the effects of shift work on DED, sleep deprivation due to shift work is considered a major mechanism. Sleep disorders can cause autonomic changes, potentially hindering tear production [5,21].

Nevertheless, the risk of DED associated with shift work by this mechanism was significantly higher only in younger subjects. Generally, aging impacts changes in the conjunctiva, meibomian glands, and lacrimal gland functional units [22]. These mechanisms impair the health of ocular surfaces, leading to a high risk of DED at older ages. In other words, in older subjects, the risk of DED due to age effects can be considered more important than the effects of shift work. On the contrary, in younger subjects, where age effects on changes in eye health are not major, it can be considered that the effect of shift work is prominent due to mechanisms such as sleep deprivation [23].

Like age, sex is a major risk factor for DED [17]. Sex hormone differences between women and men affect ocular surface conditions through meibomian glands, lacrimal glands, and conjunctival goblet cell density [24]. However, the role of an effect modifier in the risk of DED according to shift work was not confirmed in this study.

The influence of DED on shift work according to age group in each sex group was only significantly different in women. Changing levels of endocrine hormones in post-menopausal women contribute to the aging effects in the pathogenesis of DED. Thus, being female is considered a factor that intensifies aging and causes the significant difference between age groups [25]. It remains inconclusive, however, whether estrogen or androgen deficiencies or their imbalance impair ocular surface function. Accordingly, further studies are needed [25].

The prevalence of DED in this study was 3.08%. Because previous reports of the prevalence of DED in sample populations have ranged from 4.33% to 34.0%, the prevalence of DED identified in this study is less than in previous research [24,26]. The reported prevalences of DED vary according to the definition of DED and the age distribution in subjects within the study population. A high prevalence rate tends to be reported when the proportion of elderly subjects is high [24,27]. While reported prevalence varied according to the definition of DED, prevalence tends to be high when DED is defined as a symptom [24,27,28]. The participants in the current study were adult workers aged 20–64 years, which is younger than subjects in previous studies on dry eye disease [1,24,27,28,29]. Workers might have relatively low morbidity in comparison to the general population due to healthy worker effects [30]. In addition, this study defined DED using its diagnostic name and code in hospital records and NHIS data and did not show meaningful differences with the results of research using a similar definition of DED (5.28%) [31].

The strength of this study is that, to the best of our knowledge, this is the first nationally representative population-based study to identify a significant association between shift work and DED in younger subjects. Second, it is more accurate than previous research insofar as DED is defined based on actual diagnosis data rather than on subjective symptom questionnaires.

One limitation is that this study was conducted only with sample subjects in Korea, making it difficult to generalize findings to other countries and races. Second, this study is cross-sectional and cannot determine the causal relationship between shift work and DED. Moreover, there is also the possibility of the opposite mechanism, in which younger people tend to show faster rewards for side effects [32], so further research is needed. Third, although major risk factors were adjusted and stratified in this study, we could not adjust unmeasured variables such as genetic factors in DED to determine an association with shift work. In particular, there was no adjusted for visual displays terminals and the type of work, which are professionally important risk factors [33,34].

## 5. Conclusions

Our findings show an association between shift work and DED in younger subjects. Dry eye disease (DED) is a chronic disease that is difficult to cure, so prevention of risk factors is important. Shift work is an occupational hazard, and management decisions in the workplace are important. To prevent DED in young workers, unnecessary shift work should be reduced, and it is necessary to assess the risk of DED in shift workers.

## Figures and Tables

**Table 1:** Baseline characteristics of workers according to DED.

		Non-DED, *n* (%)	DED, *n* (%)	*p*-Value
Total		5691 (96.92)	181 (3.08)	
Age group (years)				
	20–39	1963 (97.95)	41 (2.05)	0.0009
	40–64	3728 (96.38)	140 (3.62)	
Work schedule				
	Daytime worker	5076 (96.96)	159 (3.04)	0.0879
	Shift worker	615 (96.55)	22 (3.45)	
Sex				
	Male	3633 (98.00)	74 (2.00)	< 0.0001
	Female	2058 (95.06)	107 (4.94)	
Occupational classification				
	White collar	2067 (97.04)	63 (2.96)	0.9318
	Pink collar	1180 (96.80)	39 (3.20)	
	Green collar	295 (96.41)	11 (3.59)	
	Blue collar	2136 (96.91)	68 (3.09)	
	Soldier or no information on job	13 (100.00)	0 (0.00)	
Smoking status				
	Never-smoker and ex-smoker	3791 (96.10)	154 (3.90)	< 0.0001
	Current smoker	1900 (98.60)	27 (1.40)	
Alcohol use				
	No	954 (96.36)	36 (3.64)	0.2688
	Yes	4737 (97.03)	145 (2.97)	
Connective tissue disease				
	No	5659 (96.95)	178 (3.05)	0.0595
	Yes	32 (91.43)	3 (8.57)	
DM				
	No	5448 (97.08)	164 (2.92)	0.001
	Yes	243 (93.46)	17 (6.54)	
Acne				
	No	5673 (96.92)	180 (3.08)	0.4489
	Yes	18 (94.74)	1 (5.26)	
Gout				
	No	5652 (96.95)	178 (3.05)	0.1385
	Yes	39 (92.86)	3 (7.14)	

DED, dry eye disease; DM, diabetes mellitus.

**Table 2:** Odds ratio (OR) of dry eye disease according to shift work by age group and sex.

	Crude OR (95% CI)	Adjusted OR ^a^ (95% CI)	*p*-Value for Interaction
Shift worker			
Overall	1.142 (0.707–1.758)	1.202 (0.741–1.860)	
Subgroup for age			
<40 years	2.323 (1.031–4.736)	3.061 (1.336–6.395)	0.037
≥40 years	0.845 (0.451–1.452)	0.923 (0.490–1.598)	
Subgroup for sex			
Male	1.507 (0.787–2.676)	1.431 (0.742–2.563)	0.449
Female	1.054 (0.487–2.013)	1.093 (0.502–2.111)	

^a^ Adjusted odds ratio was calculated by multiple logistic regression analysis after adjusting for diabetes mellitus, connective tissue disease, smoking, alcohol, acne, gout, and age. In the subgroup for sex, sex is not adjusted.

## Data Availability

Publicly available datasets were analyzed in this study. These data can be found here: [https://knhanes.cdc.go.kr/knhanes/sub03/sub03_02_05.do] (accessed on 29 March 2021).

## Appendix A

**Table A1:** Odds ratio (OR) of dry eye disease according to shift work, by age group in each sex group.

	Crude OR (95% CI)	Adjusted OR ^a^ (95% CI)	*p*-Value for Interaction
Shift worker			
Male			
<40 yeras	2.647 (0.719–8.025)	2.907 (0.782–8.933)	0.3047
≥40 years	1.184 (0.606–2.116)	1.141 (0.516–2.256)	
Female			
<40 yeras	2.760 (0.901–7.003)	3.545 (1.132–9.349)	0.0336
≥40 years	0.586 (0.204–1.330)	0.584 (0.174–1.463)	

^a^ Adjusted odds ratio was calculated by multiple logistic regression analysis after adjusting for diabetes mellitus, connective tissue disease, smoking, alcohol, acne, gout, and age. In the subgroup for sex, sex is not adjusted.

**Table A2:** Odds ratio (OR) of dry eye disease according to shift work, by age group and sex including 65 years of age or older.

	Crude OR (95% CI)	Adjusted OR ^a^ (95% CI)	*p*-Value for Interaction
Shift worker			
Overall	1.050 (0.678–1.560)	1.127 (0.725–1.683)	
Subgroup for age group			
<40 yeras	2.323 (1.031–4.736)	3.061 (1.336–6.395)	0.0242
≥40 years	0.817 (0.475–1.317)	0.934 (0.539–1.515)	
Subgroup for sex			
Male	1.350 (0.757–2.256)	1.380 (0.771–2.321)	0.4523
Female	0.972 (0.470–1.794)	1.068 (0.513–1.994)	

^a^ Adjusted odds ratio was calculated by multiple logistic regression analysis after adjusting for diabetes mellitus, connective tissue disease, smoking, alcohol, acne, gout, and age. In the subgroup for sex, sex is not adjusted.
